# Enzymatic and molecular characterisation of leucine aminopeptidase of *Burkholderia pseudomallei*

**DOI:** 10.1186/1471-2180-13-110

**Published:** 2013-05-17

**Authors:** Siew Mun Liew, Sun Tee Tay, Savithiri D Puthucheary

**Affiliations:** 1 Department of Medical Microbiology, Faculty of Medicine, University of Malaya, Kuala Lumpur, 50603, Malaysia; 2 Medical Education, Research & Evaluation Department, Duke-Nus Graduate Medical School Singapore, 8 College Road, Singapore, 169857, Singapore

**Keywords:** Leucine aminopeptidase, *Burkholderia pseudomallei*, PCR-RFLP

## Abstract

**Background:**

Leucine aminopeptidase (LAP) has been known to be a housekeeping protease, DNA-binding protein and repressor or activator in the operon regulation of virulence-associated genes in several bacterial species. LAP activity was consistently detected in overnight cultures of *Burkholderia pseudomallei,* the causative agent of melioidosis and this enzyme was partially purified and characterised in this study. The intra- and inter-species nucleotide and deduced amino acid sequence variation of LAP encoding gene (*pep*A) was determined. A *pep*A/PCR-RFLP assay was designed to facilitate the identification of major LAP sequence types amongst clinical and environmental isolates of *B. pseudomallei*.

**Results:**

LAP activity was detected in *B. pseudomallei* culture supernantants by zymographic analysis. Optimum activity was at pH 9 and stable at 50°C. Enhanced enzymatic activity was observed in the presence of metallic ions Mg^2+^, Ca^2+^, Na^+^ and K^+^. LAP activity was inhibited by EDTA, 1,10-phenanthroline, amastatin, Mn^2+^ and Zn^2+^. Sequence analysis of the complete nucleotide and deduced amino acid sequences of LAP-encoding (*pep*A) gene showed close genetic relatedness to *B. mallei* (similarity 99.7%/99.6%), but not with *B. thailandensis* (96.4%/96.4%). Eight *pep*A sequence types were identified by comparison with a 596 bp DNA fragment encompassing central regions of the *pep*A gene. A *pep*A/PCR-RFLP was designed to differentiate *pep*A sequence types. Based on restriction analysis with *Stu*I and *Hinc*II enzymes of the amplified *pep*A gene, clinical and environmental isolates showed different predominant RFLP types. Type I was the most predominant type amongst 73.6% (67/91) of the clinical isolates, while Type II was predominant in 55.6% (5/9) of the environmental isolates.

**Conclusions:**

This study showed that LAP is a secretory product of *B. pseudomallei* with features similar to LAP of other organisms. Identification of major LAP sequence types of *B. pseudomallei* was made possible based on RFLP analysis of the *pep*A gene. The high LAP activity detected in both *B. pseudomallei* and *B. thailandensis*, suggests that LAP is probably a housekeeping enzyme rather than a virulence determinant.

## Background

Bacterial enzymes have been known to play a major role in the pathogenesis of *Burkholderia pseudomallei,* the causative agent of melioidosis. This bacterium is known to secrete a number of enzymes such as protease, catalase, peroxidase, superoxide dismutase, phosphatase and phospholipase C (lecithinase), which are said to contribute to the virulence of the organism [1,2].

In our previous study [1], high levels of leucine aminopeptidase (LAP) enzymatic activity had been detected in both clinical and environmental isolates of *B. pseudomallei,* by APIZYM analysis (bioMérieux, Marcy l’Etoile, France). LAP which belongs to the peptidase M17 family, is involved in the processing and regular turnover of intracellular proteins by catalyzing the removal of unsubstituted N-terminal amino acids from various peptides [3,4]. Besides proteolytic activities, this enzyme is also known to play an important role as a DNA-binding protein in *Escherichia coli*[5], and a repressor or activator in the operon regulation of virulence-associated genes in *E. coli*, *Vibrio cholerae* and *Pseudomonas aeruginosa*[6-8]. The LAP enzyme has been proposed as an immunoantigen for vaccination against *Fasciola hepatica* in sheep [9,10] and a promising drug target for *Helicobacter pylori* infections [11].

As there has not been any study on LAP of *B. pseudomallei,* the objective of the present study was to characterise the LAP activity of *B. pseudomallei* and to examine the intra- and inter-species variation in the nucleotide and deduced amino acid sequences of the LAP encoding gene (*pep*A). A *pep*A/PCR-RFLP was designed to facilitate the identification of LAP sequence types and for possible differentiation of phenotypically identical *B. pseudomallei* isolates.

## Methods

### Extraction of LAP

One milliliter of an overnight-culture of *B. pseudomallei* NCTC 13178 (McFarland 3) was inoculated into 3 liters of BHI broth and incubated at 37°C for 72 h with constant agitation at 120 rpm in a shaker (DAIKI SCIENCES Co., Ltd., Korea). The bacterial cells were removed by centrifugation at 4,500 rpm for 30 min at 4°C, and the flow-through filtered using a 0.2 μm polyethersulfone membrane (Sartorius Stedium Biotech, Germany). One part of the filtrate was mixed with 2 parts of cold saturated ammonium sulfate solution for 10 min with stirring, prior to centrifugation at 12,000 rpm for 45 min at 4°C. The precipitate was dissolved in cold 50 mM Tris-HCl buffer (pH 7.6). Desalting was performed using HiPrep 26/10 desalting column (GE Healthcare Bio-Sciences, Sweden) coupled to a AKTA™ explorer 100 system (GE Healthcare Bio-Sciences, Sweden). The eluent was concentrated using a Vivaspin 15R column (MWCO 5,000 molecular cut-off, Sartorius Stedium Biotech, Germany) by centrifugation at 6,000 g. The protein concentration of the sample was determined by Quick Start™ Bradford Protein Assay (Bio-Rad, US) using bovine serum albumin as the standard.

### Zymographic analysis

Zymographic analysis was performed to detect the presence of LAP activity in the crude extract of *B. pseudomallei* NCTC 13178. The extract was diluted 40 fold (0.64 mg/ml) and mixed with NativePAGE™ buffer (4 X) (Invitrogen Corporation, Carlsbad) in a ratio of 3:1. The sample was analysed by native polyacrylamide gel electrophoresis (PAGE) using a one mm-thick gel (10 × 7.5 cm, 4% stacking gel and 8% resolving gel) in a Mini-PROTEAN® Tetra Cell (Bio-Rad Laboratories, US) PAGE apparatus at 90 V for 120 min. The gel was incubated at 37°C for 10 min in 50 mM Tris-HCl buffer (pH 8.0) containing 0.5 mM MgCl_2_ and 200 μM L-leucine-7-amido-4-methylcoumarin•HCl (Sigma Chemical Co., USA) dissolved in 0.5 ml acetone [12]. Five microliters of 20 X aminopeptidase I from *Streptomyces griseus* (Sigma Chemical Co., USA) was used as positive control for LAP. A fluorescent band similar to the control, representing LAP activity was visualised under UV light and photographed.

### Enzymatic characterisation

LAP activity of the crude extract was quantitated as described by Wahid *et al.*[13]. Eighty microliters of the extract was added to 20 μl of 10 mM L-leucine-p-nitroanilide substrate solution (Sigma Chemical Co., USA) and 100 μl of 50 mM Tris-HCl buffer (pH 7.6) in a microtiter well, followed by incubation at 37°C for 2 h. The reaction was stopped by cooling the mixture on ice for 10 min and the optical density at 405 nm was measured using a microplate reader (Rayto Life and Analytical Sciences Co., Ltd., China). The LAP activity was quantitated by using a L-leucine-p-nitroaniline (*p-*NA) calibration curve and defined as nanomoles of *p*-NA released per minute per milliliter of sample under the assay conditions.

The optimum pH for LAP activity was determined by incubating 80 μl of the concentrated bacterial extract with 100 μl of 50 mM buffer solutions prepared at various pHs: 6.0–7.0 (sodium phosphate buffer), 7.0–9.0 (Tris-HCl buffer), 9.0–11.0 (carbonate buffer) and 11.0–13.0 (glycine buffer). Eighty microliters of the concentrated crude extract was mixed thoroughly with 100 μl buffer of various pH in a microtiter well at 30°C for 10 min, before addition of 20 μl of substrate solution. The mixtures were incubated at 37°C for 2 h and the LAP activity was determined as described above.

The effect of temperature on LAP activity was studied by incubating for 2 h, 80 μl of the concentrated bacterial extract with 100 μl of 50 mM Tris-HCl buffer (pH 7.6) and 20 μl of 10 mM L-leucine-p-nitroanilide substrate solution at different temperatures (8, 15, 20, 30, 37, 40, 50, 60 and 80°C). The effect of metallic ions and other inhibitors on the LAP activity was investigated by exposing 80 μl of the extract to 10 μl of solution containing metallic ions (Mn^2+^, Zn^2+^, Ca^2+^, Mg^2+^, K^+^ and Na^+^), ethylenediaminetetraacetic acid (EDTA) (Amresco Inc., USA), 1,10-phenanthroline (Sigma Chemical Co., USA), phenylmethylsulfonyl fluoride (PMSF) and amastatin (AppliChem GmbH, Germany) (Table 1) and 90 μl of 50 mM Tris-HCl buffer (pH 7.6). Each mixture was pre-incubated at 30°C for 30 min before addition of 20 μl of the substrate solution. Following further incubation at 37°C for 2 h, the LAP activity of each reaction was determined as described above.

**Table 1:** **Effect of metallic ions and inhibitors on LAP activity of *B. pseudomallei *NCTC 13178**

**Compound**	**Concentration**	**Relative activity (%)**
**Control**		100
Mn^2+^	10 mM	52.2
Zn^2+^	10 mM	42.8
Ca^2+^	10 mM	126.0
Mg^2+^	10 mM	135.8
K^+^	10 mM	107.2
Na^+^	10 mM	118.0
EDTA	2 mM	0
	10 mM	0
1,10-phenanthroline	2 mM	0
	10 mM	0
Phenylmethylsulfonylfluoride (PMSF)	2 mM	69.9
	10 mM	35.9
Amastatin	2 mM	0

### Sequence determination and analysis of LAP gene

PCR primers [pepA273-F (5′-TTTCAGCCAGAAAGCCTACG-3′) and pepA1202-R (5′-GAGAAGAGGCCGGTGTTGT-3′)] were designed using computer software Primer3 (v.0.4.0) (http://frodo.wi.mit.edu/primer3/input.htm) and *T*m calculation for oligos (BioMath Calculator, Promega) (http://www.promega.com/a/apps/biomath/index.html?calc=tm) for amplification of a 930 bp fragment encompassing the central region of the *pep*A gene, using sequences retrieved from *B. pseudomallei* reference strains: 1106a [GenBank: CP000572], K96243 [GenBank: BX571965], 668 [GenBank: CP000570], 1710b [GenBank: CP000124] and MSHR346 [GenBank: CP001408] and 17 different pulsotypes of *B. pseudomallei* from a previous study [14]. Pure colonies of *B. pseudomallei* on LB agar were suspended in 500 μl MiliQ water, heated to 100°C for 30 min and cooled in ice for 10 min before centrifugation at 13,000 rpm for 10 min. The clear supernatants were used as DNA templates for amplification. Each PCR reaction was performed by preparing a 25 μl reaction mixture containing 0.25 μM of primers pepA273-F and pepA1202-R, 0.20 mM of dNTP, 1.25 U/μl of DreamTaq™ DNA polymerase (Fermentas, Lithuania), 1 X DreamTaq™ buffer, 16.63 μl of dH_2_O and 5 μl of template DNA. PCR conditions were: one cycle at 95.0°C for 5 min, and 30 cycles at 95.0°C for 1 min, 61.1°C for 30 s, 72.0°C for 1.5 min, followed by one cycle of final extension at 72.0°C for 5 min. The PCR products were purified using GeneAll® Expin™ Combo GP (GeneAll Biotechnology, Korea) and sequenced using primers pepA273-F, pepA1202-R, pepA442-F (5′-TTCACGCAGATGAAGAGCAG-3′) and pepA1037-R (5′-TTCATGCTCGTGACGATGT-3′) in an Applied Biosystems ABI3730XL automatic sequencer. The contigs of *pep*A gene sequences were assembled and edited using Geneious Pro 4.7.6 (available from http://www.geneious.com/) and aligned using Mega 4.0.2 software.

### RFLP analysis of LAP gene fragments

A PCR-RFLP assay was designed based on the *pep*A sequences. A total of 91 randomly selected clinical isolates of *B. pseudomallei* from Malaysia and 9 environmental isolates (4 from Singapore and 5 from Thailand) and 5 *B. thailandensis* isolates were used. In Additional file 1: Table S1 shows the origins of the *B. pseudomallei* isolates. Partial fragments (596 bp) of *pep*A gene were amplified from each isolate using primers pepA442-F and pepA1037-R using PCR conditions as described above, except for a higher annealing temperature of 63.9°C. The amplified products were purified and subjected to digestion using *Stu*I followed by *Hinc*II restriction endonucleases (Fermentas, Lithuania). The RFLP products were analysed by non-denaturing PAGE using 12% polyacrylamide gel and stained with 0.5 μg/ml ethidium bromide. An O’GeneRuler™ Ultra Low Range DNA ladder (Fermentas, Lithuania) was used as molecular weight marker.

## Results and discussion

**Figure 1:**
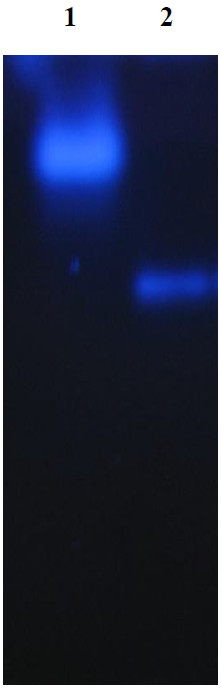
**Zymographic analysis of *B. pseudomallei *leucine aminopeptidase **[12]**.** (8% polyacrylamide gel, 8 V/cm, 120 min.). Lane 1- commercial aminopeptidase I of *Streptomyces griseus.* Lane 2- concentrated crude extract of *B. pseudomallei* NCTC 13178; *figure prints in black and white.

**Figure 2:**
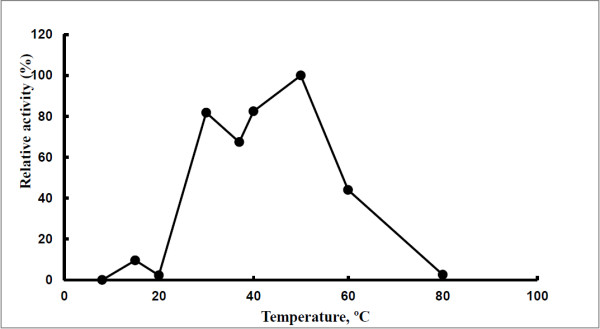
**Effect of temperature on LAP activity of *B. pseudomallei *NCTC 13178.** (activities expressed relative to maximum value).

**Figure 3:**
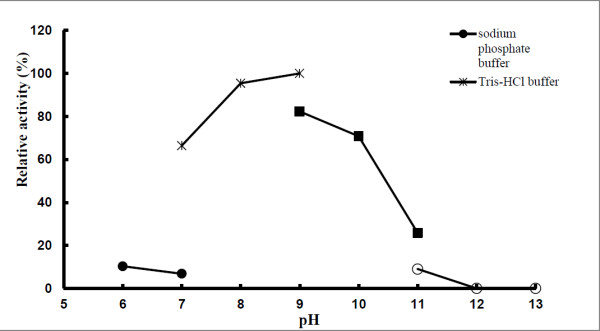
**Effect of pH on LAP activity of *B. pseudomallei *NCTC 13178.** (activities expressed relative to maximum value).

The *pep*A gene of *B. pseudomallei* consists of 1512 nucleotides and encodes for 503 amino acids. The predicted molecular mass of the expressed protein was 52.7 kD (Gene annotation). In the zymographic analysis, a fragment with fluorescent activity was observed in the native gel loaded with the concentrated culture supernatant of *B. pseudomallei* NCTC 13178 (Figure 1). The enzyme activity was detected in the culture supernatant, suggesting that LAP is a bacterial secretory product, detectable at temperatures ranging from 30°C to 60°C (Figure 2) and pH ranging from 7 to 11 (Figure 3). The optimal LAP activity was at pH 9 and at 50°C. High optimum temperature has been reported for other LAPs: i.e. 60°C for tomatoes, *E. coli* and swine [15] and 70°C for *Arabidopsis*[16], whereas the alkaline pH of LAP has been reported for organisms such as *E. coli* and *Arabidopsis thaliana*[15,16]. The alkaline pH is said to facilitate the interaction between unprotonated N-terminus substrate and hydrophobic core of LAP in order to hydrolyse the substrate [17,18]. The optimum activity of LAP at high temperature and pH (as shown in this study) may be an essential factor for *B. pseudomallei* to be extremely adaptable in a wide variety of environments and able to survive during nutritional deprivation and exposure to high temperature [19].

The effects of metal ions and inhibitors on LAP activity are shown in Table 1. There was enhancement of LAP activity in the presence of metal ions, in the order of Mg^2+^ > Ca^2+^ > Na^+^ > K^+^. This observation is in agreement with previous studies whereby a broad range of metal-ion dependence has been demonstrated by metallo-aminopeptidases: i.e. Mn^2+^ by LAPs of *E. coli*[16], Mn^2+^ by human cytosolic aminopeptidase [20] and Ca^2+^ by *Streptomyces griseus*[21]. In contrast, EDTA, 1,10-phenanthroline and amastatin inhibited LAP activity completely whereas Mn^2+^ and Zn^2+^ exhibited partial inhibitory effects (relative activities of 52.2% and 42.8% respectively). Inhibition by chelating agents (EDTA and 1,10-phenanthroline) is common in animal, plant and prokaryotic LAPs [16,22-26]. The inhibitory effects exerted by the chelating agents are suggestive that the enzyme is a metalloprotease. Amastatin which mimics the LAP catalytic transition state, is a strong inhibitor of animal aminopeptidases [25]. Hence, inhibition of LAP activity by this specific aminopeptidase inhibitor- amastatin, confirmed the identity of this enzyme as an aminopeptidase, as also described for LAP of *Streptomyces hygroscopicus*[23]. The LAP enzyme is probably not a serine protease as little impact was observed by the addition of serine protease inhibitor PMSF (only 30.1% inhibition activity was observed in this study).

Comparison of the nucleotide sequences of the central region of the *pep*A gene (596 bp) of *B. pseudomallei* reference strains: 1106a [GenBank: CP000572], K96243 [GenBank: BX571965], 668 [GenBank: CP000570], 1710b [GenBank: CP000124] and MSHR346 [GenBank: CP001408] and 17 pulsotypes of Malaysian isolates of *B. pseudomallei* revealed 8 LAP sequence types (see Additional file 1: Table S2). Nucleotide polymorphism was found at 7 positions: 465, 549, 630, 665, 685, 897 and 952, of which two at positions 549 and 685 are being reported for the first time. Examination of the deduced amino acid sequences of the enzyme shows three amino acid differences, i.e. position 222 in *B. pseudomallei* MSHR346; position 229 in strain 69 and position 318 in *B. pseudomallei* 1710b, strains 28 and 57.

Five sequence types were identified from the 17 different pulsotypes representing the genetic diversity of *B. pseudomallei* isolates in Malaysia: the majority (11 isolates) were identical to *B. pseudomallei* strain 1106a, and 3 to *B. pseudomallei* strain 668. Three strains (BP57, BP69 and BP28) were new sequence types (see Additional file 1: Table S2) suggesting slight differences existed in the conserved *pep*A gene sequence between isolates from Malaysia and those in the GenBank database. (See Additional file 1: Table S3) shows the comparison of the nucleotide and deduced amino acid sequences of *pep*A gene of *B. pseudomallei* (K96243, 1710b and MSHR346) with the closely related species (*B. mallei* ATCC 23344, *B. thailandendis* E264 and *B. oklahomensis* EO 147). Between *B. pseudomallei* K96243 and *B. thailandensis* E264, there was only 96.4% similarity in the nucleotide sequences. Comparison of 3 *B. pseudomallei* strains K96243, 1710b, MSHR346 and *B. mallei* ATCC 23344 showed only one amino acid difference. However, comparison of *B. pseudomallei* strain K96243 with *B. thailandensis* and *B. oklahomensis* showed 15 amino acid differences.

**Figure 4:**
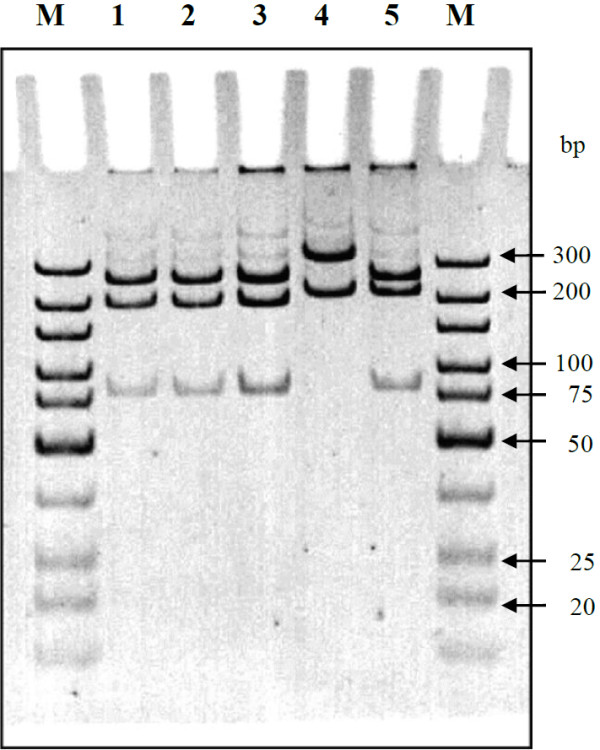
**Electrophoretic analysis of partial *pep*A gene (596 bp) of *B. pseudomallei *digested with *Stu*I and *Hinc*II restriction analysis.** (12% polyacrylamide gel, 1X TBE buffer, 8 V/cm, 130 min); Lane M- O’GeneRuler™ ultra low range DNA ladder; Lane 1- *B. pseudomallei* NCTC 13178; Lane 2- *B. pseudomallei* ATCC 23343; Lane 3- Type I; Lane 4- Type II; Lane 5- Type III.

Restriction analysis using *Stu*I and *Hinc*II of the amplified *pep*A gene enabled the identification of 3 restriction fragment polymorphism patterns (assigned as type I to III) for *B. pseudomallei*: i.e. type I with fragments of 279, 213, 83 and 20 bp; type II with fragments of 362 and 233 bp and type III with fragments of 279, 233 and 83 bp (Figure 4). Type I (73.6%) and type II (55.6%) *pep*A/RFLP types were predominant amongst our clinical and environmental isolates, respectively (see Additional file 1: Table S4).

## Conclusions

To the best of our knowledge there are no published reports on the presence or characterization of LAP in *B. pseudomallei.* DNA sequencing of 17 different pulsotypes of *B. pseudomallei* isolates showed that the partial *pep*A gene sequence was highly conserved, with the detection of 2 extra intraspecific nucleotide divergences (not reported in the *B. pseudomallei pep*A gene sequences of GenBank). We describe here the characteristics of *B. pseudomallei* LAP: high optimum temperature (50°C), alkaline optimum pH (ranging from pH 7.0 to 10.0), requirement of divalent metal ions (Mg^2+^, Ca^2+^, Mn^2+^ and Zn^2+^) for activity, and inhibition by LAP-specific inhibitors (EDTA, 1,10-phenanthroline and amastatin) and some metal ions (Mn^2+^ and Zn^2+^). The high LAP activity detected in both *B. pseudomallei* and *B. thailandensis* in both previous [1] and this study, suggests that LAP is probably a housekeeping enzyme rather than a virulence determinant. However, to verify whether LAP is truly a housekeeping gene, the use of a deletion mutant of LAP from *B. pseudomallei* will be needed. In addition, since iron is often correlated with virulence phenotypes, the effect of iron on the LAP activity should be determined. Further work to clone and express LAP as a recombinant protein is ongoing.

## Abbreviations

LAP: Leucine aminopeptidase; pepA: LAP encoding gene; PCR-RFLP: Polymerase chain reaction restriction fragment length polymorphism; PFGE: Pulse-field gel electrophoresis; BHI: Brain heart infusion; EDTA: Ethylenediaminetetraacetic acid; DNA: Deoxyribonucleic acid; APIZYM: Analytical Profile Index (ZYM); NCTC: National Collection of Type Cultures; ATCC: American Type Culture Collection; PAGE: Polyacrylamide gel electrophoresis; UV: Ultraviolet; p-NA: L-leucine-p-nitroaniline; PMSF: Phenylmethylsulfonyl fluoride; LB: Luria Bertani; dNTP: Deoxynucleotriphosphate; dH2O: Distilled water; bp: Base pair.

## Competing interests

The authors declare that there is no conflict of interests.

## Authors’ contributions

This study was carried out as part of research work for Master of Medical Science degree. All authors read and approved the final manuscript.

## Supplementary Material

Additional file 1: Table S1 Source and origin of clinical and environmental isolates of B.pseudomallei (n=100). **Table S2.** Sequence types of the *pep*A gene of *B. pseudomallei*. **Table S3.** Comparison of nucleotide and deduced amino acid sequences of *pep*A genes of *B. pseudomallei* and closely related species. **Table S4.** PCR-RFLP of partial *pep*A gene (596 bp) of *B. pseudomallei*. Click here for file
