# Supplementary material for: Enzymatic and molecular characterisation of leucine aminopeptidase of Burkholderia pseudomallei
Source: BMC Microbiol. 2013 May 17;13:110. doi: 10.1186/1471-2180-13-110 (PMC3680066; doi:10.1186/1471-2180-13-110)
Supplement: Additional file 1: Table S1 — Source and origin of clinical and environmental isolates of B.pseudomallei (n=100). Table S2. Sequence types of the pepA gene of B. pseudomallei. Table S3. Comparison of nucleotide and deduced amino acid sequences of pepA genes of B. pseudomallei and closely related species. Table S4. PCR-RFLP of partial pepA gene (596 bp) of B. pseudomallei. [file 1471-2180-13-110-S1.docx]

Additional file 1.

**Table S1. Source and origin of clinical and environmental isolates of *B. pseudomallei* (*n*=100).**

| *B. pseudomallei* | ***pep*A/RFLP Type** | | | |
| --- | --- | --- | --- | --- |
|  | **Type I** | **Type II** | **Type III** | **TOTAL** |
|  |  |  |  |  |
| **SOURCE** |  |  |  |  |
| Blood | 38 | 9 | 7 | 54 |
| Abscess (pus) | 18 | 4 | 1 | 23 |
| Urine | 2 | 1 | 0 | 3 |
| Environment | 3 | 5 | 1 | 9 |
| Unknown clinical source | 9 | 1 | 1 | 11 |
|  |  |  |  |  |
| **TOTAL** | **70** | **20** | **10** | **100** |
|  |  |  |  |  |
| **COUNTRY** |  |  |  |  |
| Malaysia | 67 | 15 | 9 | 91 |
| Thailand | 1 | 4 | 0 | 5 |
| Singapore | 2 | 1 | 1 | 4 |
|  |  |  |  |  |
| **TOTAL** | **70** | **20** | **10** | **100** |
|  |  |  |  |  |

**Table S2. Sequence types of the *pep*A gene of *B. pseudomallei.***

| **Sequence type** | **Representative strain [Genebank accession no.]** | **RFLP type** | **Malaysian strains** | **position (bp) with nucleotide variation in 1512 bp** | **Position (amino acid) with amino acid variation in 503 amino acid** |
| --- | --- | --- | --- | --- | --- |
| **1** | K96243  **[**GenBank: BX571965] | III | - | - | - |
| **2** | 668  **[**GenBank: CP000570] | III | BP33, BP42, BP125 | 399 and 630 | - |
| **3** | MSHR346  **[**GenBank: CP001408] | III | - | 630, 665 and 897 | 222 |
| **4** | 1710b  **[**GenBank: CP000124] | IV | - | 465, 630 and 952 | 318 |
| **5** | 1106a  **[**GenBank: CP000572] | I | BP91, BP7, BP9, BP14, BP53, BP55, BP71, BP72, BP73, BP92, BP112 | 465, 630, 1074, 1197 and 1326 | - |
| **6** | BP28 | II | BP28 | 549, 630 and 952 | 318 |
| **7** | BP69 | III | BP69 | 549, 630, 685 and 897 | 229 |
| **8** | BP57 | II | BP57 | 630 and 952 | 318 |

**Table S3. Comparison of nucleotide and deduced amino acid sequences of *pep*A genes of *B. pseudomallei* and closely related species.**

| ***Burkholderia* species** | ***B. pseudomallei* K96243** | ***B. pseudomallei* MSHR346** | ***B. mallei* ATCC 23344** | ***B. pseudomallei* 1710b** | ***B. thailandensis* E264** | ***B. oklohomensis* EO147** |
| --- | --- | --- | --- | --- | --- | --- |
| ***B. pseudomallei* K96243** | ID | 99.8/99.8 | 99.8/99.8 | 99.8/99.8 | 96.4/96.6 | -/96.4 |
| ***B. pseudomallei* MSHR346** | 99.8/99.8  (1 amino acid) | ID | 99.7/99.6 | 99.7/99.6 | 96.4/96.4 | -/96.2 |
| ***B. mallei* ATCC 23344** | 99.8/99.8  (1 amino acid) | 99.7/99.6 | ID | 99.8/99.6 | 96.4/96.4 | -/96.2 |
| ***B. pseudomallei* 1710b** | 99.8/99.8  (1 amino acid) | 99.7/99.6 | 99.8/99.6 | ID | 96.5/96.8 | -/96.6 |
| ***B. thailandensis* E264** | 96.4/96.6  (15 amino acid) | 96.4/96.4 | 96.4/96.4 | 96.5/96.8 | ID | -/96.4 |
| ***B. oklohomensis* EO147** | -/96.4  (15 amino acid) | -/96.2 | -/96.2 | -/96.6 | -/96.4 | -/ID |

Note: ID = identical

**Table S4. PCR-RFLP of partial *pep*A gene (596 bp) of *B. pseudomallei*.**

|  |  |  |  |
| --- | --- | --- | --- |
|  |  | **No. (%) *B. pseudomallei*** | |
|  |  | **Clinical**  **(*n*=91)** |  |
| **PCR-RFLP type** | **Expected length of DNA fragment (bp)** |  | **Environmental**  **(*n*=9)** |
|  |  |  |  |
| Type I | 279, 213, 83, 20 | 65 (71.4 %) | 3 (33.3 %) |
|  |  |  |  |
| Type II | 362, 233 | 15 (16.5 %) | 5 (55.6 %) |
|  |  |  |  |
| Type III | 279, 233, 83 | 11 (12.1 %) | 1 (11.1 %) |
|  |  |  |  |
|  |  |  |  |
